# Opening of the Electric Railway in Berlin

**Published:** 1881-06

**Authors:** 


### OPENING OF THE ELECTRIC RAILWAY IN BERLIN.

It is announced by telegraph that the electric street railway of Dr. Siemens, in Berlin, was opened for public travel on the 12th of May, with much success. A number of prominent officials and scientists were present. We have heretofore given accounts of the progress of the construction of this work. It is a narrow gauge elevated street railway, mounted on posts placed on the street sidewalks, something like portions of the elevated railway in New York, but on a smaller scale. The new railway is located on the outskirts of Berlin, and extends from the suburb known as Lichterfeld to Yeltow, a distance of about two miles. The passenger cars are narrow and short, carrying only fourteen passengers. There are two tracks. The cars are propelled by a dynamo-electric machine, which receives electricity through the track and a suspended cable, from an electric generator, one at each end of the line, each generator driven by a sixty-horse engine. An average speed of twenty miles an hour was expected to be realized. We shall give further particulars in future numbers of our paper.

The original electric railways, which were tried as experiments at Berlin and Dusseldorf exhibitions in 1879 and 1880, were worked by locomotives whose mechanism resembled a fixed dynamo-electrical machine. The rails of the line and the wheels of the locomotive engines were made of use to conduct the current of electricity and produce the necessary motion. The second conductor conveying the current produced by the stationary machine to the locomotive was connected with a system of brushes attached to the locomotive. These brushes touched a high-edged rail running in the middle of two other rails and insulated from the ground by a longitudinal sleeper. In practice, however, it has been found that this arrangement is exposed to serious interruptions. The wet snow, and mud which (according to the season) collects in the ordinary course of traffic upon the middle rail interfere very seriously at times with its conductive capacity. It has accordingly been determined on the Berlin electric line to conduct the current by the means of a copper wire properly insulated, and attached to pillars erected alongside the line, the current being conducted from the copper wire to the locomotive by means of contact rollers.—*Scientific American.*

